# Aqueous extract of *Platycodon grandiflorus* attenuates lipopolysaccharide-induced apoptosis and inflammatory cell infiltration in mouse lungs by inhibiting PI3K/Akt signaling

**DOI:** 10.1186/s13020-023-00721-z

**Published:** 2023-04-04

**Authors:** Yang Zhou, Tianzi Jin, Mingtong Gao, Zichen Luo, Sadaf Mutahir, Chen Shi, Tong Xie, Lili Lin, Jianya Xu, Yingzhao Liao, Ming Chen, Haishan Deng, Min Zheng, Jinjun Shan

**Affiliations:** 1 Medical Metabolomics Center, Institute of Pediatrics, Jiangsu Key Laboratory of Pediatric Respiratory Disease, Nanjing University of Chinese Medicine, Nanjing, 210023 China; 2 School of Pharmacy, Nanjing University of Chinese Medicine, Nanjing, 210023 China; 3 Wuhu Fanchang District People’s Hospital, Wuhu, 241200 China; 4 Department of Chemistry, University of Sialkot, Sialkot, 51300 Pakistan; 5 Department of Pediatrics, Shenzhen Hospital of Traditional Chinese Medicine, Nanjing University of Chinese Medicine, Shenzhen, 518033 China; 6 Jiangsu Suzhong Pharmaceutical Research Institute Co. Ltd, Nanjing, 210031 China

**Keywords:** Acute lung injury, Aqueous extract of *Platycodon grandiflorus*, Lipidomics, Network pharmacology, PI3K/Akt signaling pathway

## Abstract

**Background:**

Acute lung injury (ALI), an acute inflammatory lung disease, can cause a rapid inflammatory response in clinic, which endangers the patient's life. The components of *platycodon grandiflorum*, such as platycodins have a wide range of pharmacological activities such as expectorant, anti-apoptotic, anti-inflammatory, anti-tumor and anti-oxidant properties, and can be used for improving human immunity. Previous studies have shown that aqueous extract of *platycodon grandiflorum* (PAE) has a certain protective effect on ALI, but the main pharmacodynamic components and the mechanism of action are not clear.

**Methods:**

The anti-inflammatory properties of PAE were studied using the lipopolysaccharide (LPS)-induced ALI animal model. Hematoxylin and eosin stains were used to assess the degree of acute lung damage. Changes in RNA levels of pro-inflammatory cytokines in the lungs were measured using quantitative RT-qPCR. The potential molecular mechanism of PAE preventing ALI was predicted by lipidomics and network pharmacology. To examine the anti-apoptotic effects of PAE, TdT-mediated dUTP nick-end labelling (TUNEL) was employed to determine apoptosis-related variables. The amounts of critical pathway proteins and apoptosis-related proteins were measured using Western blotting.

**Results:**

Twenty-six chemical components from the PAE were identified, and their related pathways were obtained by the network pharmacology. Combined with the analysis of network pharmacology and literature, it was found that the phosphatidylinositol 3 kinase (PI3K)/protein kinase B (AKT) signaling pathway is related to ALI. The results of lipidomics show that PAE alleviates ALI via regulating lung lipids especially phosphatidylinositol (PI). Finally, the methods of molecular biology were used to verify the mechanism of PAE. It can be found that PAE attenuates the inflammatory response to ALI by inhibiting apoptosis through PI3K/Akt signaling pathway.

**Conclusion:**

The study revealed that the PAE attenuates lipopolysaccharide-induced apoptosis and inflammatory cell infiltration in mouse lungs by inhibiting PI3K/Akt signaling. Furthermore, our findings provide a novel strategy for the application of PAE as a potential agent for preventing patients with ALI.

**Supplementary Information:**

The online version contains supplementary material available at 10.1186/s13020-023-00721-z.

## Background

Acute lung injury (ALI) is used by a variety of extrapulmonary pathogenic factors such as serious infection, trauma toxicity and disseminated intravascular coagulation. Acute respiratory distress syndrome characterized by the formation of protein-rich fluid in the alveolar cavity can be developed at the severe end of this disease spectrum. Furthermore, it can cause acute respiratory failure with increased pulmonary vascular permeability and pulmonary edema [1–4]. Chinese medicine has accumulated rich experience in the treatment of ALI [5]. It is possible to use Chinese medicine to intervene in the treatment of ALI, which provides certain advantages for the treatment of clinical ALI.

*Platycodon grandiflorus* is the dry roots of *Platycodon grandiflorus* (Jacq.) A. DC, which is an important traditional Chinese medicine. It has the functions of moistening lungs, reducing phlegm, and expelling pus, and it is mainly used to treat symptoms such as pharyngitis, vomiting caused by purulent swelling, infection and chest pain [6]. *Platycodon grandiflorus* contains many active ingredients, such as platycodins [7]. A large number of studies have shown that platycodin has a wide range of pharmacological activities, such as expectorant, anti-apoptotic, anti-inflammatory, anti-tumour and antioxidant properties [8, 9]. Chinese medicine believes that *Platycodon grandiflorus* mainly acts on the lungs and its related parts. Previous studies have shown that aqueous extract of *Platycodon grandiflorus* (PAE) has a certain protective effect on ALI [10], but its main pharmacodynamic components and mechanism of action are still unclear.

The change of lipid composition in pulmonary surfactant is a common feature of many acute and chronic respiratory diseases such as ALI [11]. Previous studies showed that ALI can affect the lipids in the lungs of mice. The major modern method for analysing numerous complicated lipid molecules is chromatography-mass spectrometry [12]. Network pharmacology is a combination of system biology network analysis and drug pleiotropic design methods. It can be used to build a complex interaction network of drugs, drug targets and disease targets to predict the target of chemical constituents in traditional Chinese medicine and reveal the complex action mechanism of traditional Chinese medicine components [13–15]. Recently, several researchers have successfully combined metabolomics with network pharmacology to explore the interactions between organisms and drugs [16–18]. Combining lipidomics and network pharmacology offers the possibility to explore the potential mechanism of PAE for the prevention of ALI.

In this study, the molecular mechanism of the effect of active components on ALI was studied by lipidomics and network pharmacology methods. Firstly, it is proved that PAE has a certain prevention effect on ALI from the pharmacodynamics of animal experiments. Then, network pharmacology and lipidomics were used for predicting the mechanism of PAE in the prevention of ALI. Finally, the methods of molecular biology were used to verify the mechanism of PAE.

## Materials and methods

### Materials

Lipopolysaccharide (LPS, No. L2880, 055: B5) was purchased from Sigma (St. Louis, MO, USA). Dexamethasone (DXMS, No. H37021898) was purchased from Chenxin Pharmaceutical Co, Ltd (Jining, Shandong Province, China). Mouse PIP3 ELISA (enzyme-linked immunosorbent assay) Kit (No. ZC-38629) was purchased from ZCIBIO Technology Co, Ltd. *Platycodon grandiflorum* (No.101206) was purchased from the Anhui Fengyuan Tongling TCM Decoction Pieces Co, Ltd (Tongling, Anhui Province, China). Sichuan Weikeqi Biological Technology Co, Ltd provided the reference standard deapio dealio platycodin D, platycodin D2 and platycoside D. (Chengdu, Sichuan Province, China). Avanti Polar Lipids Company provided SM (17:0) (batch number: 170SM-13) and PE (17:0/17:0) (batch number: LM170PE-19) (Alabaster, AL, USA). ROE Co. provided isopropanol, MTBE, ammonium formate and ammonium acetate with a mass spectrometry purity of 99.8%. (Oradell, NJ, USA). Merck provided methanol, acetonitrile and formic acid with a mass spectrometry purity of 99.8%. (Darmstadt, Germany). Ultrapure water filtered via using a Milli-Q water purification system was used to make all aqueous solutions (Millipore, Milford, MA, USA).

### Apparatus

Qualitative analysis of PAE: ACQUITY UPLC® H-Class System (Waters Co, Milford, MA, USA) and LTQ Orbitrap XL™ hybrid FT mass spectrometer (Thermo Fisher Scientific Inc, Bremen, Germany). Lipidomics: UltiMate® 3000 ultra-high-performance liquid chromatography system (DIONEX, Sunnyvale, CA, USA) and Q Exactive™ Hybrid Quadrupole-Orbitrap™ Mass Spectrometer (Thermo Fisher Scientific Inc, Bremen, Germany).

### Preparation of PAE

Seventy-five grams of *Platycodon grandiflorus* were boiled for 1.5 h after being soaked in 0.9 L water for 30 min. After pouring out the first decoction, 0.75 L of water was added and boiled for 1 h. Finally, two decoctions were combined, filtered through gauze and concentrated to achieve PAE concentrations of 0.151, 0.3775, or 0.755 g/mL.

### Qualitative analysis of PAE

#### PAE sample pretreatment

The liquid from the preparation of PAE was diluted with water to 150 mg/mL and centrifuged at 18,000 rpm for 10 min. Then, the supernatant was filtered by a 0.22 μm membrane for LC-LTQ-Orbitrap tandem mass spectrometry qualitative detection. The reference standards used were deapio platycodin D, platycodin D_2,_ and platycoside D.

#### Chromatographic conditions

The chromatographic column and column temperatures were Hypersil GOLD C18 (100 mm × 2.1 mm, 3 μm) and 40 °C respectively. The mobile phase was composed of A (acetonitrile) and B (0.1% formic acid–water) using a gradient elution of 23% A at 0–18 min, 29% A at 18–22 min, 90% A at 22–23.5 min and 23% A at 23.5–28 min. The flow rate was set at 0.3 mL/min.

#### MS conditions

All MS experiments were performed in the negative ion modes. The source and ion transfer parameters applied were as follows: spray voltage 3.5 kV and the capillary voltage 35 V. The sheath gas, aux gas, atomization temperature, and the capillary temperature were maintained at 35 arb, 15 arb, 350 °C, and 300 °C, respectively. The tube lens level was set at 110 V and the resolution of FT was 6000.

#### Network pharmacology

Based on the qualitative analysis of PAE, the Canonical SMILES format of chemical components was downloaded from PubChem Database (https://pubchem.ncbi.nlm.nih.gov) and the targets of chemical components were selected from the SwissTargetPrediction database [19–21] (http://www.swisstargetprediction.ch/), with the species limited to “Homo sapiens” [22–24]. Furthermore, ALI-associated targets were acquired from Gene Cards Database (https://www.genecards.org/), OMIM Database (https://omim.org/,) and DisGeNET (https://www.disgenet.org/) which were searched using the keywords “Acute Lung Injury” [25, 26]. Venny 2.1 (https://bioinfogp.cnb.csic.es/tools/venny/) was used to take the intersection of PAE and ALI targets [27, 28], which were defined as potential preventive targets.

A “drug-ingredient-target-disease” regulatory network was established based on potential preventive targets by Cytoscape3.7.2 software. The protein–protein interaction (PPI) was further created based on potential preventive targets using String Database (https://string-db.org/). R version 3.6.0 and Bioconductor software packages were applied to analyze Gene Ontology (GO) and Kyoto Encyclopedia of Genes and Genomes (KEGG) pathways. Finally, the top 20 related KEGG pathways were selected for further network analysis. An integrated “target-composition-pathway” network was constructed by Cytoscape 3.7.2.

#### Animals and LPS-induced ALI

C57BL/6 J male mice [license: SYXK (Su) 2018–0049] were obtained from Shandong Provincial Laboratory Animal Center (Jinan, Shandong Province, China). Before the experiment, all mice were acclimatised to the laboratory settings for three days. The mice were kept in separate cages and subjected to conventional circumstances such as 12 h light–dark cycles, 45% relative humidity and a constant temperature of 23 ± 2 °C. With a weight range of 18–22 g (8–10 weeks old), those mice were randomly divided into the following six groups (*n* = 6): Control, LPS, LPS + DXMS (10 mg/kg) and LPS + PAE (1.51, 3.775, 7.55 g/kg/d, respectively) groups. DXMS was used as a positive control. According to the Chinese Pharmacopoeia, the maximum clinical dose of *Platycodon grandiflorum* was 10 g, and the equivalent clinical dose for mice was 1.51 g/kg. The dose group set in this study was equivalent, 2.5 and 5 times the clinical dose, respectively. Studies have shown the non-toxicity of PAE in above dose [29, 30]. After 7 days, LPS, LPS + DXMS and LPS + PAE groups were induced by intratracheal administration of 1 mg/mL LPS (3 mg/kg). Firstly, mice were anesthetized with sodium pentobarbital (60 mg/kg) by intraperitoneal injection and placed in a supine position, and then LPS was injected at 3 mg/kg into the LPS, LPS + DXMS and LPS + PAE groups mice’s trachea [21]. Six hours later, all the mice were sacrificed. Biological samples and lung tissues were collected.

#### Histopathologic evaluation of the lung tissue

To assess histological alterations in the lung tissues, the tissues were fixed in 4% paraformaldehyde, then paraffin-embedded and stained with hematoxylin and eosin (H&E). A light microscope was used to examine pathological alterations in the lung tissues.

#### Reverse-transcription quantitative polymerase chain reaction (RT-qPCR)

To begin, total RNA was isolated from mouse lung tissues using Trizol reagent (Invitrogen, Carlsbad, CA, United States). Second, a one-step RT kit (Takara Biotechnology, Dalian, Liaoning Province, China) was used to reversely transcribe total RNA to first-strand cDNA for a 1 g sample. Finally, the reactions were carried out in 10-L quantities with GAPDH (Sangon Biotech, Shanghai, China) serving as an internal control. To achieve the mean value, experiments were done in triplicate for each sample. The relative expression of mRNA was determined using the 2^−ΔΔCT^ method. Apoptotic cytokines including Bcl-2 and Bax as well as inflammatory cytokines such as IL-1β, IL-6 and TNF-α were measured. The primer sequences used in this study are listed in Table 1.

**Table 1:** Primer sequences RT-qPCR

Gene	Forward primer	Reverse primer
GAPDH	AACGACCCCTTCATTGAC	TCCACGACATACTCAGCAC
IL-1β	GCAACTGTTCCTGAACTCAACT	ATCTTTTGGGGTCCGTCAACT
IL-6	CTCCCAACAGACCTGTCTATAC	CCATTGCACAACTCTTTTCTCA
TNF-α	ATGTCTCAGCCTCTTCTCATTC	GCTTGTCACTCGAATTTTGAGA
Bcl-2	GCTACCGTCGTGACTTCGC	CCCCACCGAACTCAAAGAAGG
Bax	AGACAGGGGCCTTTTTGCTAC	AATTCGCCGGAGACACTCG

### Lipidomics analysis of lung

#### Sample preparation

Lung tissues (20 mg) were weighed into a 2 mL centrifuge tube with two ball mills and 200 μL ultrapure water for homogenization. Then lung tissues homogenate (20 µL) was added to a 1.5 mL centrifuge tube followed by the addition of 225 µL ice methanol solution with the internal standard [SM (17:0) and PE (17:0/17:0)]. The concentration of the internal standard is around 5 μg/mL. For 10 s, the mixture was vortexed. Seven hundred and fifty microliters of methyl tert-butyl ether (MTBE) were added, and the mixture was shaken for 10 min at 4 °C. The samples were vortexed for 10 s before being centrifuged for 2 min at 14000 rpm at 4 °C after being added 188 L of ultrapure water. In the organic phase, mainly lipids were transferred to fresh tubes and dried in a vacuum centrifuge. Finally, the residue was reconstituted with 110 µL methanol: toluene (9:1) for analysis.

#### Lipidomic analysis

A reversed-phase Waters Acquity UPLC CSH C18 (100 mm × 2.1 mm, 1.7 µm) column was used for chromatographic separation and maintained at 65 °C. The positive ion mode mobile phase was composed of acetonitrile/water (6:4, v/v) (eluent A) and isopropanol/acetonitrile (9:1, v/v) containing 0.1% formic acid and 10 mM ammonium formate (eluent B). The mobile phase of the negative ion mode was the same as that of the positive ion mode except that no formic acid was added. Both the positive and negative ion modes used a gradient elution of 15% B at 0 min, 30% B at 0–2 min, 48% B at 2–2.5 min, 82% B at 2.5–11 min, 99% B at 11–12 min and 15% B at 12–15 min. The flow rate was set at 0.6 mL/min.

Q Exactive Hybrid Quadrupole-Orbitrap Mass Spectrometer was used for both positive and negative ion modes. Parameters of mass spectrometry: spray voltage was 3.5 kV (positive) and 3.0 kV (negative); for both ionization modes, sheath gas, aux gas, capillary temperature, and heater temperature were maintained at 35 arb, 15 arb, 325 °C and 300 °C, respectively; scan range was *m/z* 215–1800.

#### Data processing

Raw spectra files of all samples (including QC) from both ion modes were converted to ABF format by using Reifycs ABF Converter (http://www.reifycs.com/AbfConverter/). For each ion mode, ABF files were imported into MS-DIAL software to match the primary and secondary fragmentation ions with the built-in Lipid Blast database. The results exported from MS-DIAL were manually checked. The unknown annotations, annotations without MS2 information and duplicated annotations were deleted. To reduce instrumental errors and retain biological errors, the data matrix was normalized by R language. Based on the smallest RSD of QCs, the data matrix was imported into SIMCA-P 13.0 for Partial Least Squares Discriminant Analysis (PLS-DA). At the same time, R language was used to conduct a non-parametric test (Kruskal–Wallis test), and the fold change value was calculated according to the median peak height of each annotation, with criteria set at *P* < 0.05 and fold change (FC) ≥ 1.2 or ≤ 0.8333 to screen differential metabolites [32, 34]. Further, the bubble diagram was used to cluster different lipids in total ion mode by ChemRICH (http://chemrich.fiehnlab.ucdavis.edu/) and cluster analysis was performed on the different lipids in positive and negative ion modes via using a heatmap. Lastly, GraphPad Prism 8.0 was used to observe the expression of different PIs in the three groups.

#### Western blotting

Proteins were isolated from lung tissues in each group, and the total protein content was determined by using a BCA protein assay kit (Thermo Scientific, Rockford, IL, USA). Then, using SDS-PAGE, equal amounts of proteins were separated, transferred to PVDF membranes (0.45 m, Millipore, Milford, MA, USA), and blotted overnight at 4 °C with primary antibodies against PI3K (1:1000), p-PI3K (1:1000), Akt (1:1000), p-Akt (1:2000), Bcl-2 (1:1000), Bax (1:5000), and β-actin (1:10,000), followed by secondary antibodies incubation. The protein bands were quantified using the Image Lab software (Bio-Rad Co, Hercules, CA, United States) and the ChemiDoc™ MP Imaging System (Bio-Rad Co, Hercules, CA, United States). At least three times, each experiment was carried out.

#### Immunohistochemical analysis

Cross-immunohistochemical staining in paraffin sections was employed to reveal the presence of phosphorylated phosphatidylinositol 3-kinase (p-PI3K), phosphorylated protein kinase B (p-Akt), B-cell lymphoma-2 (Bcl-2), and Bcl-2 associated X protein (Bax).

#### TdT-mediated dUTP nick-end labelling (TUNEL) staining

TUNEL labelling was performed to analyze the apoptosis rate of lung cells after embedding in paraffin and cutting into 40 µm sections, as is the standard approach [35]. Fluorescence staining was carried out according to the manufacturer's instructions using Roche Diagnostics GmbH's in-situ Cell Death Detection Kit (Mannheim, Germany). A light microscope was used to view the apoptotic cells, with the results represented as the average number of TUNEL-positive stained cells per 500 magnification field.

#### Enzyme-linked immunoassay of phosphatidylinositol triphosphate (PIP3)

Solid-phase antibody was prepared by coating microporous plates with purified mouse PIP3 antibody, and then PIP3 was added in rapid succession into the microporous plates coated with monoclonal antibody. The above one was then bound with a PIP3 antibody labelled by human haptoglobin-related proteins (HRP) to form an antibody-antigen-enzyme labelled antibody complex. After thoroughly washing the complex, the substrate tetramethylbenzidine (TMB) was added to it to develop its colour. TMB was first converted into blue under the catalysis of the HRP enzyme, and then into the final yellow under the action of acid. The intensity of the colour is positively correlated with the phosphatidylinositol triphosphate (PIP3) in the sample. So, the absorbance (OD value) was measured with a microplate reader at 450 nm. The concentration of mouse phosphatidylinositol triphosphate (PIP3) in the sample was calculated from the standard curve.

## Statistical analysis

The data were presented as mean standard deviation and analyzed using GraphPad Prism 8.0’s one-way analysis of variance (ANOVA) function. *P* < 0.05 was used to determine if differences were statistically significant.

## Results

### Separation and identification of chemical components of PAE

**Fig. 1:**
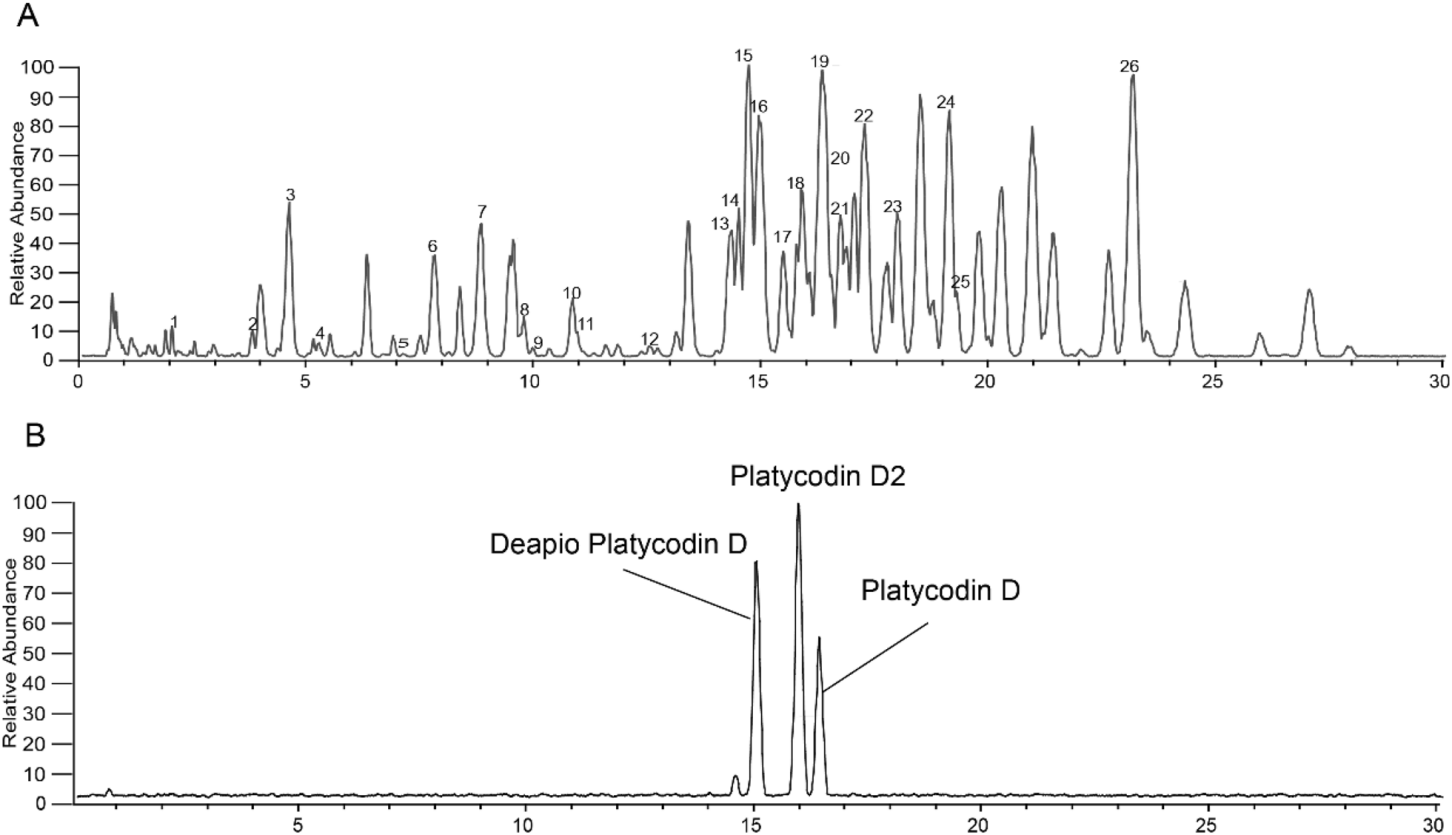
**A** Total ion flow diagram of PAE. **B** Platycodin reference under negative ion mode

A total of 26 chemical components in PAE were preliminarily identified based on the mixed reference standard solution and our in-house database. The detailed chemical information of PAE was shown in Fig. 1 and Table. 2. *Platycodon* saponin D was chosen as the marker ingredient for the quality control of PAE in this work, a reference to the quality control method for *Platycodon* in the Chinese Pharmacopoeia (2020 edition) [6]. The content, RSD and linearity (R^2^) of *Platycodon* saponin D in PAE in the experiments of this work were 1.057 mg/kg, 3.3% and 0.9982, respectively.

**Table 2:** Chemical components in the PAE for qualitative analysis

NO.	Identification	RT (min)	Experimental m/z	Formular	Error(ppm)
1	Platycoside L	2.10	843.44147	C_42_H_68_O_17_	3.096
2	Platycoside G2	3.74	1283.59497	C_59_H_96_O_30_	3.606
3	Platycoside E	4.56	1547.67603	C_69_H_112_O_8_	0.148
4	Platycoside D	5.21	1531.68469	C_69_H_112_O_7_	3.723
5	Platycoside G1	7.06	1415.63599	C_64_H_104_O_4_	2.367
6	Deapio-platycodin D3	7.73	1253.58167	C_58_H_94_O_29_	0.87
7	Platycodin D3	8.78	1385.62341	C_63_H_102_O_3_	0.352
8	Platycoside H/isomer	9.71	1237.58252 [M + HCOO]^−^ 1283.59436	C_58_H_94_O_28_	− 3.365
9	Platycoside M-1	9.90	677.35565	C_36_H_54_O_12_	1.400
10	Polygalacin D3	10.79	1369.63135	C_63_H_102_O_2_	3.206
11	Platycodon saponin 1	10.90	697.38208	C_36_H_58_O_13_	2.712
12	Platycoside G3	12.46	1385.62732	C_63_H_102_O_3_	4.262
13	Platycoside F	14.27	959.48822 [M + HCOO]^−^ 1005.49274	C_47_H_76_O_20_	2.502
14	Platycoside A	14.44	1253.58142	C_58_H_94_O_29_	0.62
15	Platyconic acid A	14.67	1237.54773	C_57_H_90_O_29_	− 1.769
16	Deapio platycodin D	14.89	1091.52563	C_52_H_84_O_24_	− 2.346
17	Platycodin D2	15.82	1385.62183	C_63_H_102_O_3_	− 1.228
18	Platycoside C	15.97	1133.53979	C_5_H_86_O_25_	2.346
19	Platycodin D	16.29	1223.56750	C_57_H_92_O_28_	− 2.735
20	Platycoside J	16.48	1075.53345 [M + HCOO]^−^ 1121.53992	C_52_H_84_O_23_	0.388
21	Polygalacin D2	16.81	1369.62842	C_63_H_102_O_2_	0.276
22	Platycodin C	17.20	1265.57861	C_59_H_94_O_29_	− 2.19
23	Polygalacin D	17.71	1207.57544	C_57_H_92_O_27_	0.12
24	2''-*O*-acetylplatyconic acid A	19.08	1279.55847	C_59_H_92_O_30_	− 1.594
25	Platycoside B	19.23	1133.53979	C_54_H_86_O_25_	2.346
26	Platycoside K	23.03	843.44147	C_42_H_68_O_17_	3.096

### *PAE exerts protective effects against LPS-induced ALI *in Vivo

**Fig. 2:**
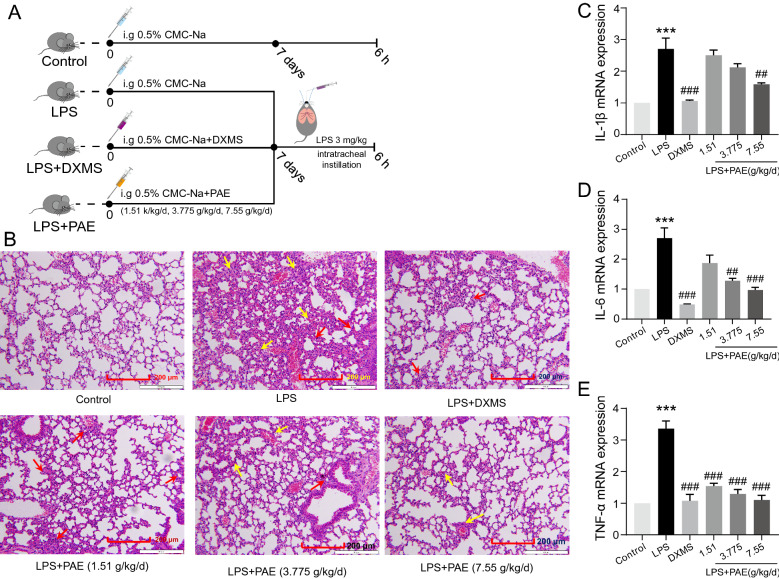
**A** Experimental scheme (*n* = 6). **B** Lung tissues sections were stained with H&E for histopathological analysis (magnification 200 × , n = 3). The black arrow marks the place where the pathology has changed. PAE reduced the mRNA expression of inflammatory cytokines, including, **C** IL-1β, **D** IL-6 and **E** TNF-α. ^***^P < 0.001 and ^****^P < 0.0001 compared with the control group; ^##^*P* < 0.01, ^###^*P* < 0.001and ^###^*P* < 0.0001 compared with the LPS group

According to the “Animals and LPS-induced ALI” section, Fig. 2A shows an experimental scheme for the induction of ALI in mice. After the mice were sacrificed, the lung tissues were used for pro-inflammatory cytokines detection and pathological changes evaluation. As expected, alveolar walls in the control group were thin, no exudation in the bronchial lumen, no inflammatory cell infiltration into the alveolar walls and around the interstitial spaces was observed. Thickening of alveolar walls, bronchial epithelial cell degeneration and necrosis, dead cells in the lumen, and a significant number of exudates and inflammatory cells may all be seen in the LPS group of mice. These LPS-induced degenerative alterations were greatly reduced by DXMS and PAE therapies (Fig. 2B). Several indicators linked to LPS-induced ALI, such as cytokines: IL-1β, IL-6, and TNF-α in lung tissues, were measured to further investigate the protective effects of PAE during experimental ALI (Fig. 2C–E). LPS-induced ALI lung edema and inflammation could be reduced by treatment with DXMS or PAE. These findings show that PAE, particularly PAE at a dosage of 7.55 g/kg/d, can prevent LPS-induced ALI by protecting the alveolar vascular barrier's integrity and limiting inflammatory cell infiltration.

### Network pharmacology predicts potential signaling pathways for PAE prevention of ALI

As a traditional Chinese medicine, PAE is characterized by multiple components, pathways and targets. We used network pharmacology to predict the targets of PAE and ALI and obtained a significant wealth of signaling pathways, which provided a direction for further study of the molecular mechanism.

**Fig. 3:**
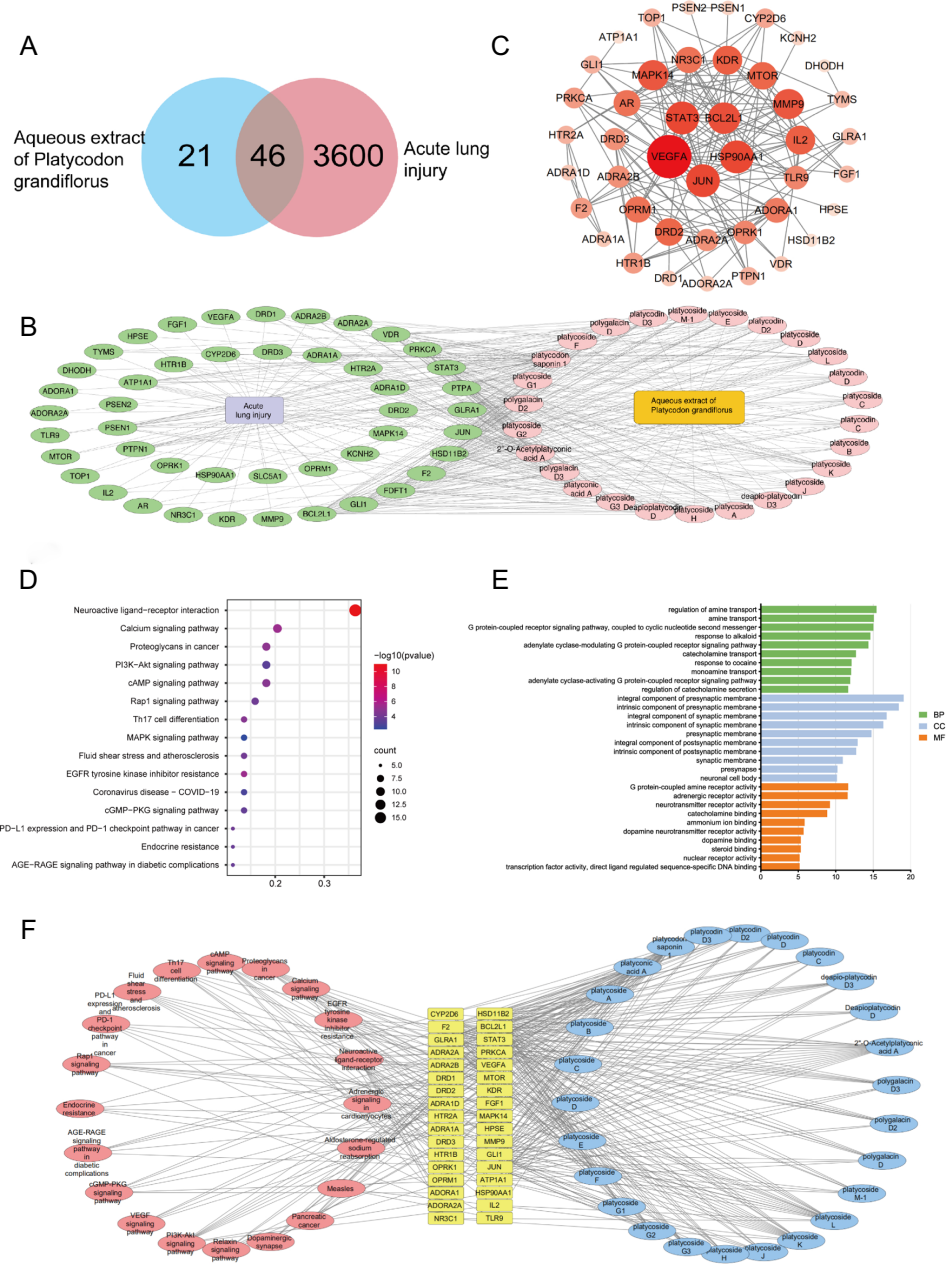
**A** Venn Fig. of 46 potential targets. **B** Drug-ingredient-target-disease network. The disease was marked in light purple, disease targets (46 in total) in green, the drug was marked in orange and chemical components (26 in total) in pink. **C** Protein interaction (PPI) network. **D** KEGG enrichment analysis of the top twenty signaling pathways. **E** The top 10 of GO enrichment analysis (biological process, molecular function, and cellular component). F Target-composition-pathway network. Compositions were marked in light blue, signaling pathways were marked in pink, and genes were marked in light yellow

Based on the databases of PubChem, SwissTargetPrediction, OMIM, DisGeNET, and GeneCards, 67 PAE-related targets and 3646 ALI-associated targets were obtained. 46 potential targets were obtained (Fig. 3A) and used for constructing the "drug-ingredient-target-disease" network (Fig. 3B). Results indicate that PAE may play a synergistic role in the prevention of ALI through multiple potential targets.

The knowledge of the interactions between proteins is the foundation for further exploring molecular mechanisms. To clarify the underlying interactions of the 46 potential targets, the PPI network was constructed in String Database (Fig. 3C) and revealed the biological functions and underlying mechanisms of PAE, KEGG enrichment analysis revealed 47 significantly enriched signaling pathways (Fig. 3D). As a result, 1133 terms in biological process (BP), 69 in cellular component (CC), and 116 in molecular function (MF) were differentially expressed by GO pathway enrichment (Fig. 3E). In addition, a target-composition-pathway network was constructed based on the results of KEGG enrichment analyses (Fig. 3F). A large number of studies have shown that PI3K/Akt signaling pathway plays an irreplaceable role in the exploration of the mechanism of ALI [31, 36–38]. The genes related to PI3K/Akt signaling pathway include p-PI3K, p-Akt, Bcl-2, etc. PI3K/Akt signaling pathway is the junction of multiple signaling pathways in vivo, and plays an important role in anti-inflammatory and anti-apoptotic [39]. Combined with Fig. 3E and literature, the intervention of PAE on ALI is related to PI3K/Akt signaling pathway.

### PAE can alleviate ALI by regulating lung lipid metabolism

The PI3K/Akt signaling pathway is a signaling pathway related to phosphatidylinositol in cells. The phospholipid is an important component of lung surface-active substance as well as the cell membrane. The metabolism of surfactant lipid is closely related to lung diseases, and the change of its composition is a common feature of many acute and chronic respiratory diseases. Therefore, the study of lipid metabolism in ALI is important.

**Fig. 4:**
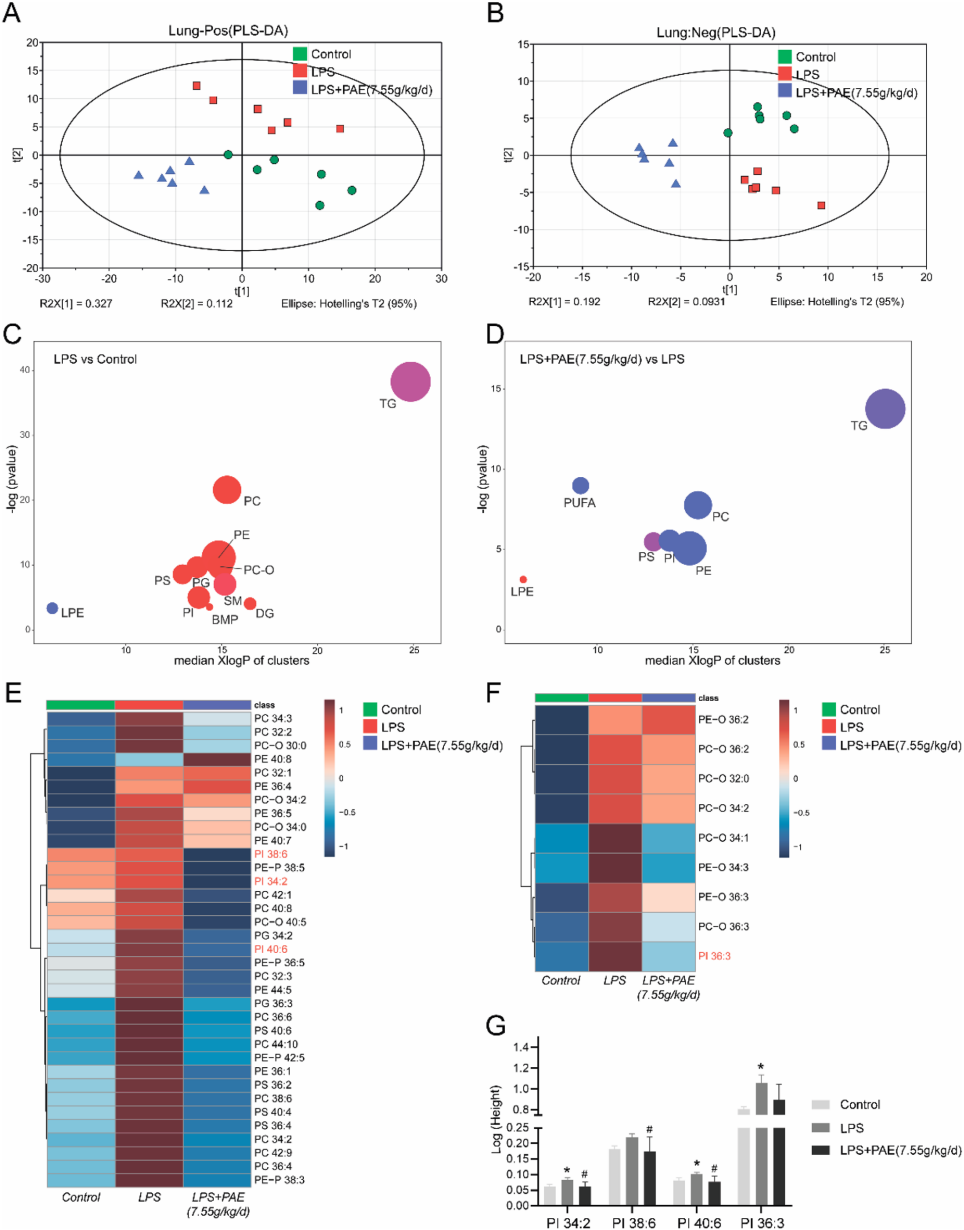
**A** and **B** Partial least squares discriminant analysis (PLS-DA) score plots of mice pulmonary lipids among control, LPS, and LPS + PAE groups. N = 6, per group: **A** positive ion mode; **B** negative ion mode. **C** and **D** ChemRICH enrichment plots of differential lipids screened in **C** LPS vs Control and D LPS + PAE (7.55 g/kg/d) vs LPS groups. The size of each circle was proportionate to the number of lipids in the same class. Red colour represented up-regulation, blue colour represented down-regulation, and purple colour represented up or down-regulation, respectively. **E** and **F** Heatmap of differential lipids that up-regulated in LPS vs Control group while downregulated in LPS + PAE (7.55 g/kg/d) vs LPS groups: **E** positive ion mode and **F** negative ion mode. Each square in the heatmap represents the corresponding average intensity value of a lipid of samples in each group, red squares represent an increase in concentration and blue ones represent a decrease in concentration.** G** The concentration of phosphatidylinositol in lung tissues of mice among control, model, and LPS + PAE (7.55 g/kg/d). ^*^P < 0.05 compared with the control group; ^#^*P* < 0.05 compared with the LPS group

To investigate the correlation between ALI and lipid metabolism, various lipid compounds were analyzed by UHPLC-Q Exactive Orbitrap mass spectrometer from samples in control, LPS and LPS + PAE (7.55 g/kg/d) groups. The PLS-DA shows a good distinction between the three groups (Fig. 4A, B). ChemRICH enrichment plots show the higher levels of PI, PE and PC in lung tissues from the LPS group, and all of them could be reversed by PAE administration (Fig. 4C, D). Additionally, the heatmap further confirmed the changing trends of lipids that were up-regulated in ALI and down-regulated after medication (Fig. 4E, F). As shown in histograms, PI was highly abundant in the LPS group, while they were decreased dramatically in the LPS + PAE (7.55 g/kg/d) group (Fig. 4G). It shows that PAE can regulate lung lipid, especially PI to alleviate ALI. This provides a basis for the prevention of ALI by PAE.

### PAE prevents ALI by inhibiting PI3K/Akt signaling pathway

**Fig. 5:**
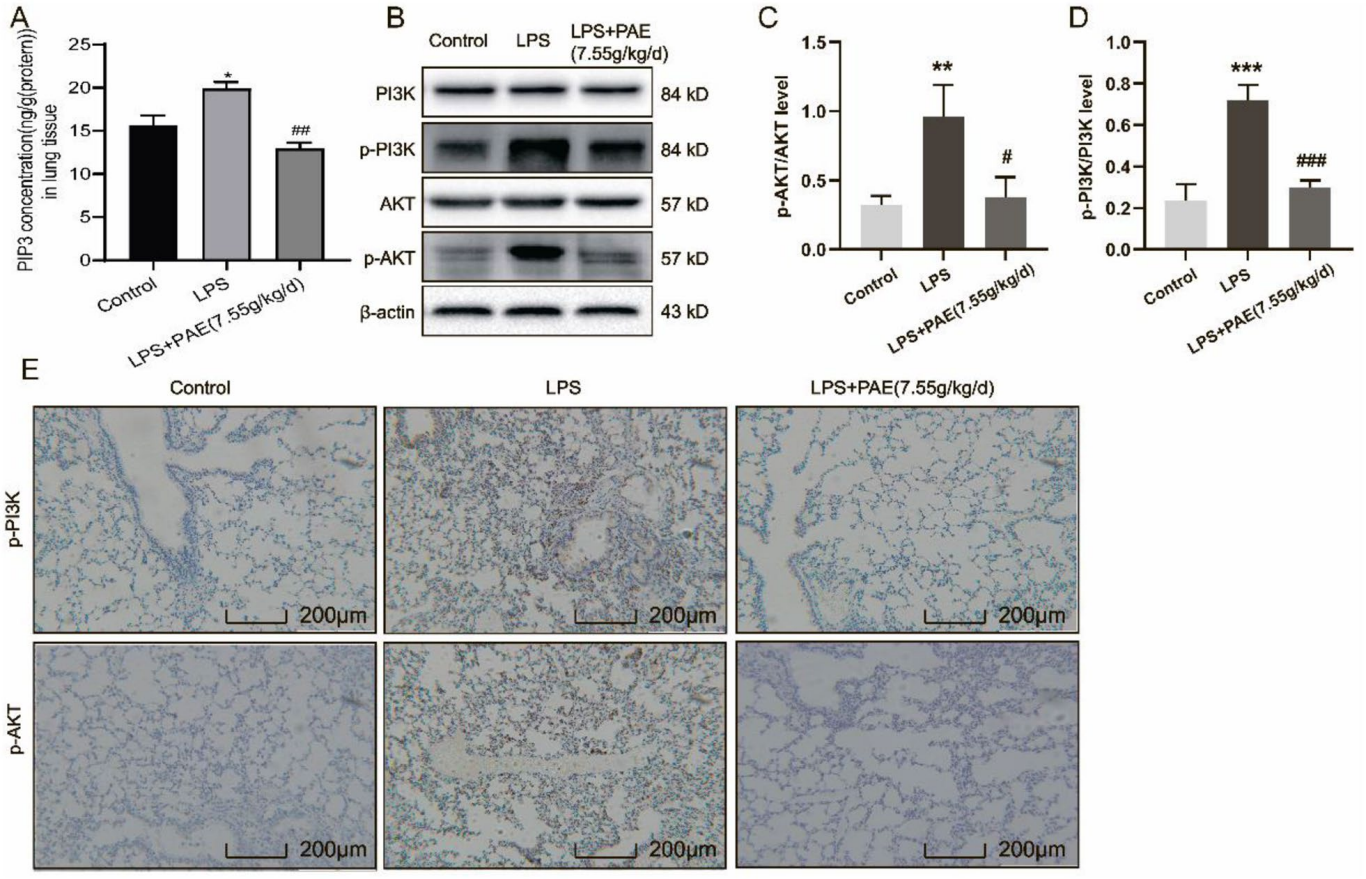
**A** ELISA analysis of PIP3 expression in lung, *n* = 3. B Western blot analysis of p-PI3K and p-Akt levels in lung. Representative images of **C** p-AKT and **D** p-PI3K protein levels in lung determined by western blot, *n* = 3. **E** Immunohistochemistry staining of p-PI3K and p-Akt protein (magnification 200 ×). ^**^P < 0.01 and ^**^P < 0.001 compared with the control group; ^#^*P* < 0.05 and ^###^*P* < 0.001 compared with the LPS group

Through lipidomics and network pharmacological methods, it is predicted that the PI3K/Akt pathway might be the main prevention pathway of PAE and speculate that PAE may alleviate ALI by reducing PI to inhibit PI3K/Akt signaling pathway. Therefore, the level of PIP3, which was generated and accumulated by PI3K to recruit protein kinase B (Akt) and active Akt, was measured by ELISA. The related genes such as p-PI3K and p-Akt, which are the major markers of the activated PI3K-Akt signaling pathway, were measured by immunohistochemical analysis and western blotting. As shown in Fig. 5A–D, LPS + PAE (7.55 g/kg/d) group downregulated the PIP3, p-PI3K, and p-Akt levels in LPS-induced ALI mice compared with the LPS group. Besides, Fig. 5E shows that p-PI3K and p-Akt positive areas in the LPS + PAE (7.55 g/kg/d) group were significantly reduced compared to the LPS group. These results suggest that the PI3K/Akt signaling pathway should be activated in ALI, and PAE might prevent ALI by reducing PI to inhibit the PI3K/Akt signaling pathway.

### PAE inhibits apoptosis by regulating levels of apoptosis-related proteins in ALI

**Fig. 6:**
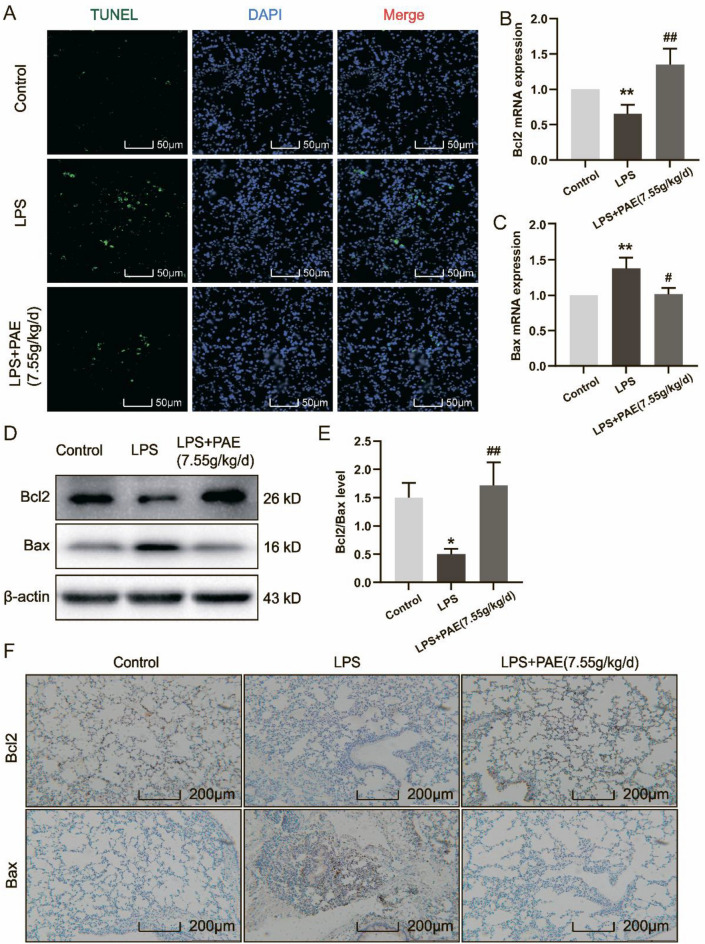
**A** From left to right: TUNEL staining, DAPI staining and merge staining (magnification 500 ×). Statistical results of mRNA expressions of **B** Bcl-2 and **C** Bax, *n* = 3. **D** Western blot analysis of Bcl-2 and Bax levels in lung. **E** Statistical results of Bcl-2/Bax protein level in lung determined by western blot, *n* = 3. **F** Immunohistochemistry staining of Bcl-2 and Bax protein (magnification 200 ×). ^**^P < 0.01 compared with the control group; ^#^*P* < 0.05, ^##^*P* < 0.01 and ^###^*P* < 0.001 compared with the LPS group

PAE may inhibit the PI3K/Akt signaling pathway to prevent ALI. This signaling pathway also involves apoptosis-related genes, e.g., Bax and Bcl-2, which are the major markers of activated apoptosis. To determine whether PAE reduced apoptosis in ALI mice, TUNEL staining was employed to quantify the number of apoptotic cells in lung tissue (Fig. 6A), and the expression levels of Bcl-2 and Bax in lung tissue were determined by RT-qPCR, Western blotting and immunohistochemical staining (Fig. 6B–F).

As expected, the TUNEL staining results showed that the number of apoptotic cells was significantly increased in the LPS-treated group, and the expression levels of Bcl-2 and Bax in the corresponding lung tissues were significantly decreased and increased, respectively. However, PAE treatment led to a significant decrease in the number of apoptotic cells, an increase in the expression level of Bcl-2, and a decrease in the expression level of Bax. These findings suggest that PAE should prevent ALI via inhibiting apoptosis in the lungs of LPS-induced ALI mice.

## Discussion

ALI is a clinical syndrome characterized by pulmonary inflammation and alveolar-capillary barrier destruction, which is often secondary to severe pneumonia sepsis, severe trauma and other diseases. A variety of inflammatory cells are involved in the occurrence and development of ALI pulmonary inflammation [40]. At present, symptomatic treatment is the main clinical treatment with no effective treatment addressing the basic mechanisms of ALI. Therefore, it is urgent to find new effective treatment methods for ALI. Different from most synthetic chemical medicine, traditional Chinese medicine provides a multi-component, multi-channel, and multi-targeted [41] therapy that has some advantages for the treatment of ALI in the clinic. LPS is a common causative factor of ALI, and the LPS-induced ALI model was used to study the prevention of PAE on ALI [40, 42]. Through the experiment, it was proved that PAE could protect against LPS-induced ALI by reducing pulmonary oedema and the growth of inflammatory cytokines. However, understanding the in vivo molecular mechanism of the anti-apoptosis and anti-inflammatory effects of PAE remains a key point.

Network pharmacology analysis of 26 active ingredients of PAE in ALI showed 67 target proteins of these components, including Bcl-2, PTPA, VEGFA, etc. These target proteins are associated with ALI. Bcl-2 protein is related to the positive regulation of the metabolic process of human reactive oxygen, B cell proliferation, and internal apoptotic signaling pathways [43, 44]. PTPA protein has a significant inhibitory effect on cytokines related to the NF-κB signaling pathway in the early stage of immunity, especially in the early stage of signaling activation, which is significantly related to the regulation of human immunity and inflammation [45]. The increase in apoptosis and inflammation reflects the degree of lung tissue damage in patients with ALI [46]. This provides some ideas and directions for us to study the prevention mechanisms of ALI by PAE. The 46 potential targets between PAE targets and ALI ones were used to further analyze the signaling pathways involved in the prevention of ALI by PAE. Combined some papers find that PI3K/Akt signaling pathway was mostly associated with LPS-induced ALI [47–49]. In membrane lipid metabolism, PI3K phosphorylates PI-4-P (PIP) produce PI-3,4-P_2_ (PIP2) and PIP2 produces PI-3,4,5-P_3_ (PIP3). These membrane-bound PIP3 provide anchoring sites for a variety of signal transduction proteins, which in turn mediate a variety of downstream signaling pathways. The PI3K/Akt signaling pathway is a signaling pathway related to phosphatidylinositol in cells [50, 51]. These give us a way to link lipids and signaling pathways. Pulmonary surfactant is a lipid-proten complex secreted by alveolar type II epithelial cells, of which approximately 90% are lipids [52]. The lipid is an important component of cells, participating in the life activities of the body and providing energy for the body [51]. Previous studies have shown that LPS can cause disturbance in the metabolism of lung lipids, especially phospholipids [34, 53]. The changes in lipids in the lung showed that several PIs were upregulated in the LPS group, while treatment of PAE (7.55 g/kg/d) provided a callback effect on PIs in the lung tissues. This indicates that PAE could regulate the lung lipid levels, especially PI to alleviate ALI. Combining the results of network pharmacology and lipidomics, we predicted that the PI3K/Akt signaling pathway is one of the molecular mechanisms. Previous reviews have shown that phosphatidylinositol-3-kinases (PI3K) is a lipid kinase, which generates the second messenger phosphatidylinositol-3,4,5-trisphosphate (PIP3) and accumulates the PIP3 to recruit protein kinase B (Akt) and active Akt. Akt activation plays an important role in inflammation, apoptosis, and immunity [50, 54]. The correlation between inflammatory response and PI3K/Akt signaling pathway had been reported in several other papers [37, 55, 56]. Therefore, to study the relationship between PI3K/Akt signaling pathways and ALI, we studied the PI3K/Akt signaling pathway and its downstream apoptosis-related pathways related to proteins via molecular biology experiments, such as PIP3, and p-PI3K, p-Akt, Bcl-2, Bax, etc.

The data showed that ALI could activate the PI3K/Akt signaling pathway to play a series of injuring roles. However, one may notice that the research reports on the PI3K/Akt signaling pathway were controversial in the studies of LPS-induced acute lung injury (ALI). There is still no clear result on whether the activation or inhibition of the PI3K/Akt signaling pathway on the protective effect of ALI [47–49, 57–59]. Readers may find the details about the relationship between PI3K/Akt signaling pathway and ALI in the Additional file. In these studies, such as references [47–49, 58], the PI3K/Akt signaling pathway was activated in ALI. On the contrary, it was reported in references [57, 59] that the PI3K/Akt signaling pathway was inhibited in ALI. The role of the PI3K/Akt signaling pathway in ALI remains to be studied further. It is interesting to note that in cell experimental studies [60], it was found that the PI3K/Akt signaling pathway was dose-dependent with the dose of LPS. Low-dose PI3K/AKT signaling pathway was excited, while the high-dose PI3K/Akt signaling pathway was inhibited, providing a certain research direction for LPS-induced ALI in PI3K/Akt signaling pathway studies.

## Conclusion

In summary, it was systematically revealed via lipidomics and network pharmacology that PAE can effectively alleviate the ALI-induced apoptosis and decrease the inflammatory response. Our preliminary conclusion is that PAE can be used for preventing ALI by inhibiting the PI3K/Akt signaling pathway and a good regulating effect on lipid metabolism disorder caused by LPS-induced ALI, which lays a foundation for a certain direction for the clinical treatment of ALI. Furthermore, the effect of ALI on the PI3K/AKT signaling pathway is an issue that needs to be explored.

## Supplementary Information

**Additional file 1: Table S1.** A summary of recent studies on the PI3K/AKT signaling pathway in ALI (animal experiments).

## Data Availability

The research data generated from this study are included in the article and Additional files.

## Abbreviations

Akt: Protein kinase B
ALI: Acute lung injury
ANOVA: Analysis of variance
Bax: Bcl-2-associated X protein
BP: Biological process
Bcl-2: B-cell lymphoma 2
CC: Cellular component
DXMS: Dexamethasone
ELISA: Enzyme-linked immunosorbent assay
HRP: Dexamethasone human haptoglobin-related proteins
IL: Interleukin
LPS: Lipopolysaccharide
MF: Molecular function
MTBE: Methyl tert-butyl ether
PI: Phosphatidylinositol
PI3K: Phosphatidylinositol3-kinase
PIP3: Phosphatidylinositol triphosphate
PAE: Aqueous extract of *platycodon grandiflorum*
RT-qPCR: Reverse-transcription quantitative polymerase chain reaction
TMB: Tetramethylbenzidine
TNF: Tumor necrosis factor
TUNEL: TdT-mediated dUTP nick-end labeling
