# Supplementary material for: Aqueous extract of Platycodon grandiflorus attenuates lipopolysaccharide-induced apoptosis and inflammatory cell infiltration in mouse lungs by inhibiting PI3K/Akt signaling
Source: Chin Med. 2023 Apr 4;18:36. doi: 10.1186/s13020-023-00721-z (PMC10071731; doi:10.1186/s13020-023-00721-z)
Supplement: Supplementary file 1 — Additional file 1: Table S1. A summary of recent studies on the PI3K/AKT signaling pathway in ALI (animal experiments). [file 13020_2023_721_MOESM1_ESM.docx]

**Additional file**

**Table S1** A summary of recent studies on the PI3K/AKT signaling pathway in ALI (animal experiments)

| Studies | Model (Activation or Inhibition) |
| --- | --- |
| [1] | Inhibition |
| [2] | Inhibition |
| [3] | Activation |
| [4] | Activation |
| [5] | Activation |
| [6] | Inhibition |
| [7] | Inhibition |
| [8] | Activation |
| [9] | Activation |
| [10] | Inhibition |
| [11] | Inhibition |
| [12] | Inhibition |
| [13] | Inhibition |
| [14] | Activation |
| [15] | Activation |
| [16] | Activation |
| [17] | Activation |
| [18] | Inhibition |
| [19] | Inhibition |
| [20] | Inhibition |

**References**

[1] Huang Y, Chen S, Pang L, Feng Z, Su H, Zhu W, et al. Isovitexin protects against acute liver injury by targeting PTEN, PI3K and BIP via modification of m6A. European Journal of Pharmacology. 2022; 917: 174749.

[2] Zhong R, Xia T, Wang Y, Ding Z, Li W, Chen Y, et al. Physalin B ameliorates inflammatory responses in lipopolysaccharide-induced acute lung injury mice by inhibiting NF-κB and NLRP3 via the activation of the PI3K/Akt pathway. Journal of Ethnopharmacology. 2022; 284: 114777.

[3] Yang B, Wang R, Ji LL, Li XP, Li XH, Zhou HG, et al. Exploration of the function of ginsenoside Rd attenuates lipopolysaccharide-induced lung injury: a study of network pharmacology and experimental validation. Shock. 2022; 57 (2): 212-220.

[4] Chen H, Li N, Zhan X, Zheng T, Huang X, Chen Q, et al. Capsaicin protects against lipopolysaccharide-induced acute lung injury through the HMGB1/NF-κB and PI3K/AKT/mTOR pathways. Journal of Inflammation Research. 2021; 14: 5291-5304.

[5] He X, Wu J, Tan T, Guo W, Xiong Z, Yang S, et al. Quassinoids from Brucea javanica and attenuates lipopolysaccharide-induced acute lung injury by inhibiting PI3K/Akt/NF-κB pathways. Fitoterapia. 2021; 153: 104980.

[6] Wang L, Tang X, Li S. Propofol promotes migration, alleviates inflammation, and apoptosis of lipopolysaccharide-induced human pulmonary microvascular endothelial cells by activating PI3K/AKT signaling pathway via upregulating APOM expression. Drug Devel Research. 2022; 83 (2): 397-406.

[7] Fei X, Zi QY, Bing WY, Min L, Xin MX, Zhen M, et al. Aldosterone alleviates lipopolysaccharide-induced acute lung injury by regulating epithelial sodium channel through PI3K/Akt/SGK1 signaling pathway. Molecular and Cellular Probes. 2021; 57: 101709.

[8] Huang J, Nong X, Chen Y, Zhang A, Chen L. 3-O-trans-caffeoyloleanolic acid improves acute lung injury via anti-inflammation and antioxidative stress-involved PI3K/AKT pathway. Chemical Biology Drug Design. 2021; 98 (1): 114-126.

[9] Liu JH, Li C, Cao L, Zhang CH, Zhang ZH. Exosomal miR-132-3p from mesenchymal stem cells alleviated LPS-induced acute lung injury by repressing TRAF6. Autoimmunity. 2021; 54 (8): 493-503.

[10] Xu HR, Yang Q, Xiang SY, Zhang PH, Ye Y, Chen Y, et al. Rosuvastatin enhances alveolar fluid clearance in lipopolysaccharide-induced acute lung injury by activating the expression of sodium channel and Na, K-ATPase via the PI3K/AKT/Nedd4-2 pathway. Journal of Inflammation Research. 2021; 14: 1537-1549.

[11] Zhang Q, Wang Z, Zhu J, Peng Z, Tang C. Ferulic acid regulates miR-17/PTEN axis to inhibit LPS-induced pulmonary microvascular endothelial cells apoptosis through activation of PI3K/Akt pathway. The Journal of Toxicological Sciences. 2022; 47 (2): 61-69.

[12] Jiang Y, Xia M, Xu J, Huang Q, Dai Z, Zhang X. Dexmedetomidine alleviates pulmonary edema through the epithelial sodium channel (ENaC) via the PI3K/Akt/Nedd4-2 pathway in LPS-induced acute lung injury. Immunologic Research. 2021; 69 (2):162-175.

[13] Tang QY, Wei JX, Xue SF, Liu GH, Fu LX. Fibrogrowth factor-2 protects against acute lung injury by activating the PI3K/Akt signaling pathway. Journal of Biological Regulators and Homeostatic Agents. 2020; 34 (5): 1679-1688.

[14] An L, Zhao J, Sun X, Zhou Y, Zhao Z. S-allylmercaptocysteine inhibits mucin overexpression and inflammation via MAPKs and PI3K-Akt signaling pathways in acute respiratory distress syndrome. Pharmacological Research. 2020; 159: 105032.

[15] Li R, Zou X, Huang H, Yu Y, Zhang H, Liu P, et al. HMGB1/PI3K/Akt/mTOR signaling participates in the pathological process of acute lung injury by regulating the maturation and function of dendritic cells. Frontiers In Immunology. 2020; 11: 1104.

[16] Ma C, Zhu L, Wang J, He H, Chang X, Gao J, et al.Anti-inflammatory effects of water extract of taraxacum mongolicum hand-mazz on lipopolysaccharide-induced inflammation in acute lung injury by suppressing PI3K/Akt/mTOR signaling pathway. Journal of Ethnopharmacology. 2015; 168: 349-55.

[17] Huang CY, Deng JS, Huang WC, Jiang WP, Huang GJ. Attenuation of lipopolysaccharide-induced acute lung injury by hispolon in mice, through regulating the TLR4/PI3K/Akt/mTOR and Keap1/Nrf2/HO-1 pathways, and suppressing oxidative stress-mediated ER stress-induced apoptosis and autophagy. Nutrients. 2020; 12 (6): 1742.

[18] Ding Z, Zhong R, Yang Y, Xia T, Wang W, Wang Y, et al. Systems pharmacology reveals the mechanism of activity of Ge-Gen-Qin-Lian decoction against LPS-induced acute lung injury: A novel strategy for exploring active components and effective mechanism of TCM formulae. Pharmacological Research. 2020; 156: 104759.

[19] Zhang L, Ge S, He W, Chen Q, Xu C, Zeng M. Ghrelin protects against lipopolysaccharide-induced acute respiratory distress syndrome through the PI3K/AKT pathway. Journal of Biological Chemistry. 2021; 297 (3): 101111.

[20] Luo X, Lin B, Gao Y, Lei X, Wang X, Li Y, et al. Genipin attenuates mitochondrial-dependent apoptosis, endoplasmic reticulum stress, and inflammation via the PI3K/AKT pathway in acute lung injury. International Immunopharmacology. 2019; 76: 105842.
